# Supplementary material for: Brain connectivity during Alzheimer’s disease progression and its cognitive impact in a transgenic rat model
Source: Netw Neurosci. 2020 Apr 1;4(2):397–415. doi: 10.1162/netn_a_00126 (PMC7286303; doi:10.1162/netn_a_00126)
Supplement: Supplementary file 1 [file netn-04-397-s001.pdf]

Muñoz-Moreno, E., Tudela, R., López-Gil, X., & Soria, G. (2020). Supporting information for "Brain connectivity during Alzheimer's disease progression and its cognitive impact in a transgenic rat model." *Network Neuroscience*, 4(2), 397–415. https://doi.org/10.1162/netn_a_00126

Supplementary Materials

Index

1. Network-based statistic results
2. Figures
3. Tables

1. Network-based statistic results

Networks altered in TgF344-AD animals resulting from NBS analysis involved the following regions:

- FD-w connectome at 8 months of age:
  - Right hemisphere: accumbens, superior colliculus, midline dorsal and ventromedial thalamus, temporal association cortex, medial geniculate and caudate putamen.
  - Left hemisphere: corpus callosum and cingulate cortex.
  - Both hemispheres: insular cortices.
- FA-w connectome at 15 months of age:
  - Right hemisphere: accumbens, frontal association cortex and cingulate cortex
  - Left hemisphere: corpus callosum, auditory cortex, anterior commissure, piriform cortex, visual cortex, posterior hippocampus, hippocampus subiculum, periaqueductal gray and superior colliculus.
  - Both hemispheres: entorhinal cortex, insular cortex, orbitofrontal cortex, caudate putamen, parietal association and somatosensory cortex, motor cortex, amygdala, IPAC and restrosplenial cortex.
- Functional connectome at 15 months of age:
  - Right hemisphere: midline dorsal and ventromedial thalamus, olfactory tubercle and visual cortex.
  - Left hemisphere: parietal association and somatosensory cortex.

2. Figures

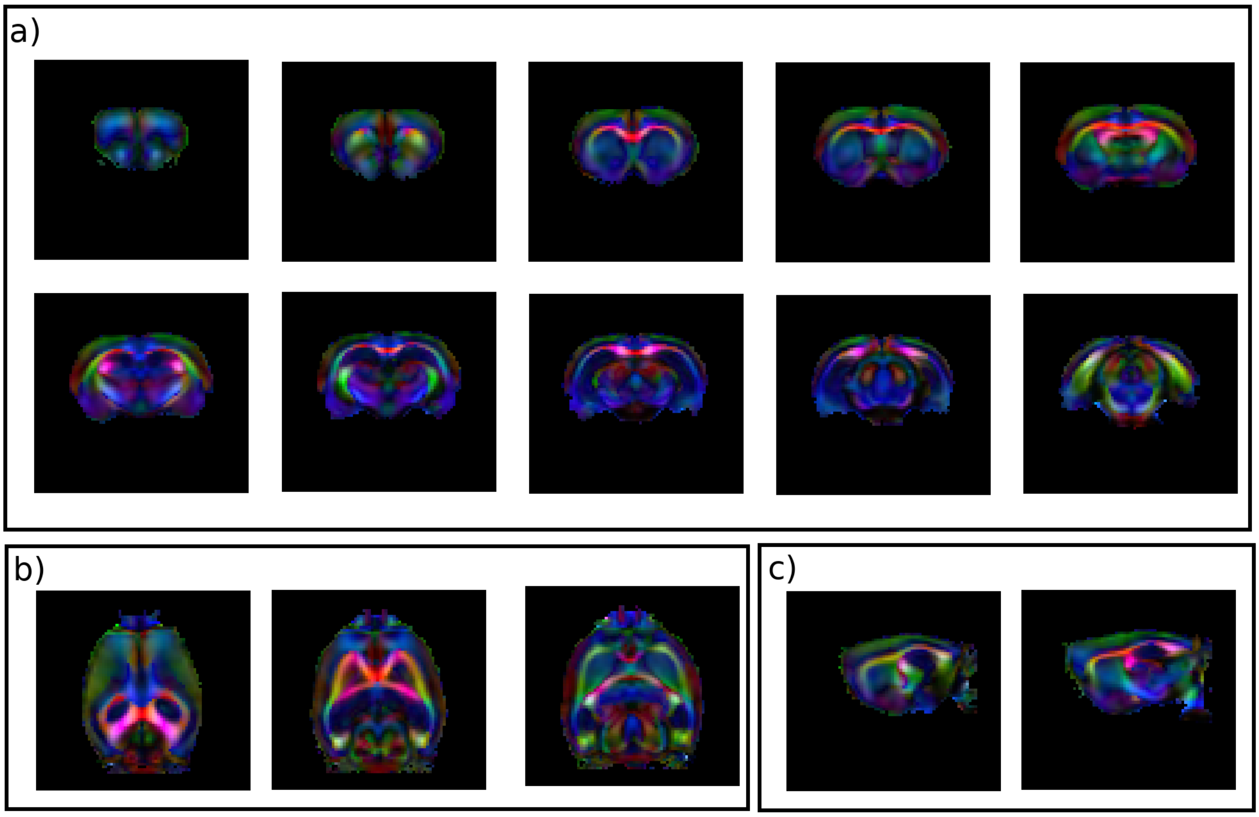

Supplementary Figure 1. Representative slices of colored fractional anisotropy of one of the diffusion acquisitions. Color code: red, right-left direction; green, ventral-dorsal direction; blue: rostral-caudal direction. a) coronal slices from anterior to posterior; b) axial slices from superior to inferior; c) sagittal slices from left to right (left hemisphere).

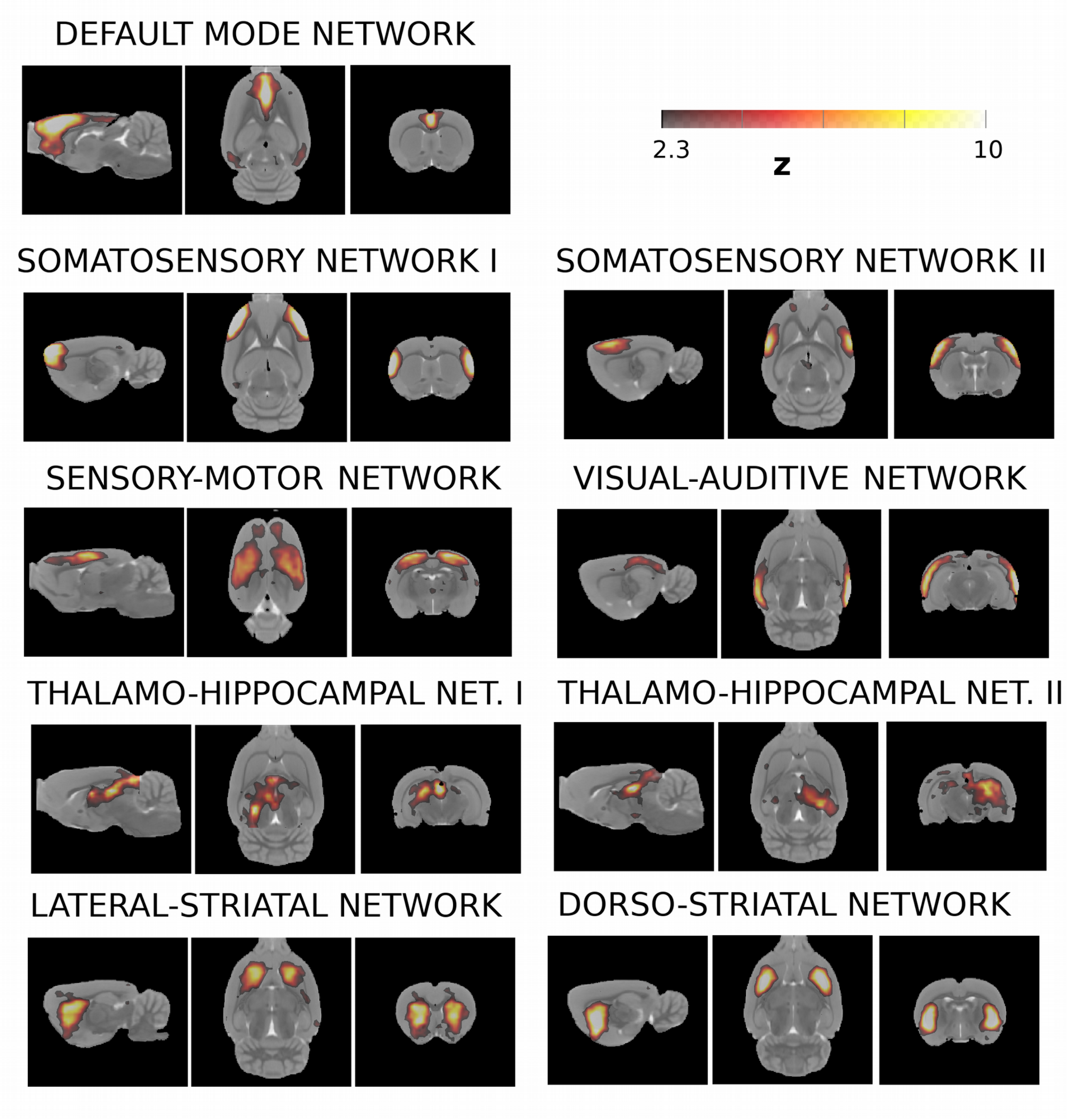

Supplementary Figure 2. Representative slices of networks obtained by independent component analysis (ICA) of the dataset. ICA was performed using FLS's MELODIC (Jenkinson, Beckmann, Behrens, Woolrich, & Smith, 2012). The analysis was set to extract 30 components. The 9 networks shown in this figure were selected based on its correspondence to networks described in literature (Bajic, Craig, Borsook, Becerra, & Sullivan, 2016; Hsu et al., 2016; Sierakowiak et al., 2015).

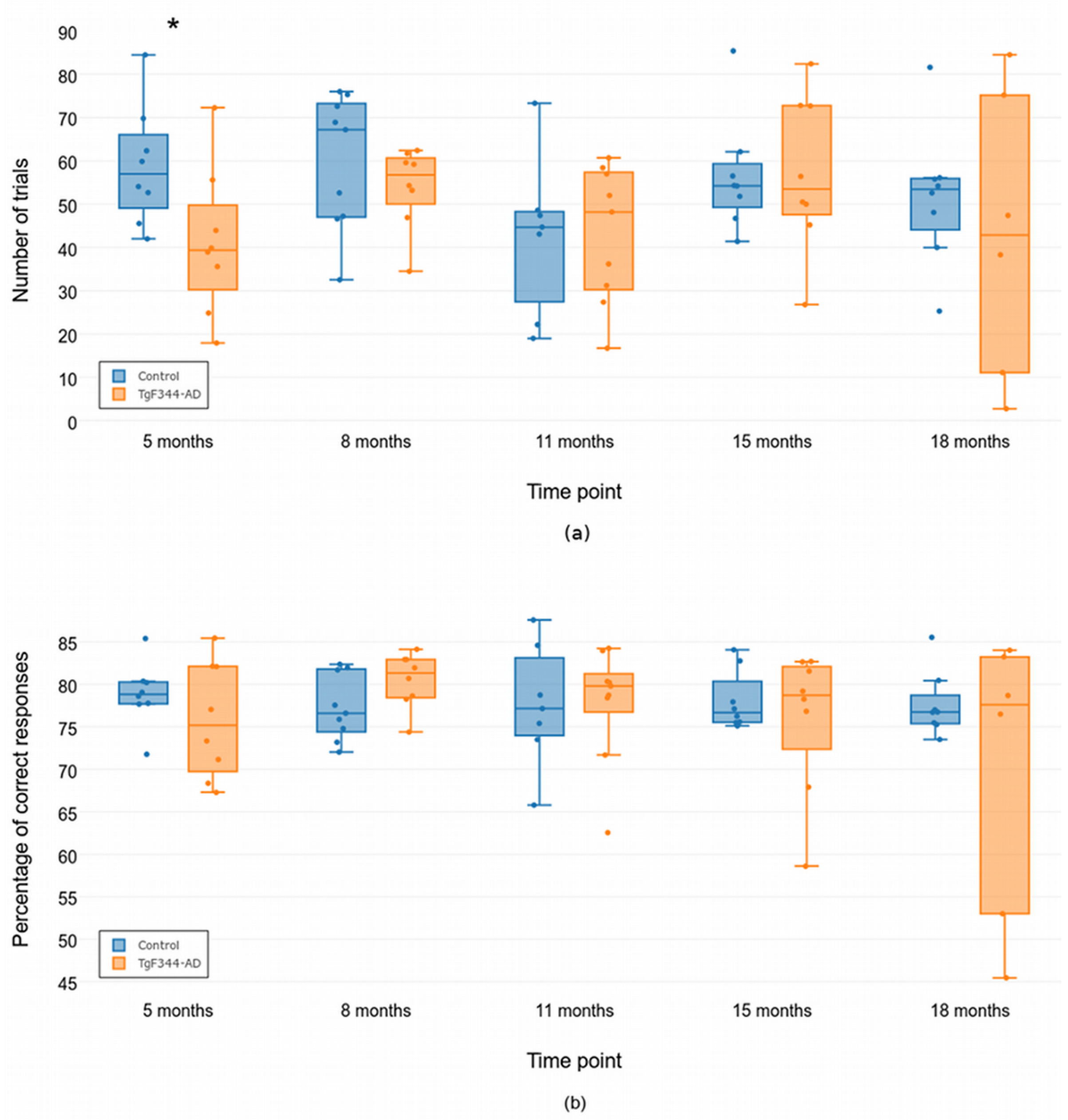

Supplementary Figure 3. Cognitive performance. (a) Average number of trials per DNMS session and (b) percentage of correct responses in the DNMS task in the five evaluated time points (blue: control group; orange: TgF344-AD group). Asterisk represents uncorrected $p<0.05$. No significant differences were observed after FDR correction.

3. Tables

		Group		Age		Group-age interaction	
**Connectome**	**Network metric**	**p_{FDR}**	**Cohen's f^2**	**p_{FDR}**	**Cohen's f^2**	**p_{FDR}**	**Cohen's f^2**
FA-weighted	Strength	0.0084*	0.2553**	0.0285*	0.2143**	0.0946	0.5226***
	Global efficiency	0.1164	0.2271**	0.1149	0.1284*	0.5259	0.1004*
	Local efficiency	0.3012	0.1934**	0.4528	0.0425*	0.9151	0.0039
	Clustering coeff.	0.0266*	0.2838**	0.0400*	0.2112**	0.2772	0.2984**
FD-weighted	Strength	0.1607	0.2029**	0.1088	0.1209*	0.0323*	0.8118***
	Global efficiency	0.4155	0.1958**	0.3323	0.0528*	0.1378	0.4246***
	Local efficiency	0.2479	0.2135**	0.1469	0.0961*	0.0460*	0.7060***
	Clustering coeff.	0.1164	0.1491*	0.0673	0.1536**	0.0276*	0.9047***
Structural binary	Degree	0.0084*	0.2305**	0.0285*	0.2383**	0.0276*	0.9597***
	Global efficiency	0.0084*	0.2305**	0.0285*	0.2383**	0.0276*	0.9599***
	Local efficiency	0.0084*	0.2186**	0.0083*	0.3353**	0.0276*	0.9154***
	Clustering coeff.	0.0084*	0.2186**	0.0083*	0.3352**	0.0276*	0.9154***
Functional weighted	Strength	0.9056	0.0154	0.4716	0.0280*	0.9151	0.0071
	Global efficiency	0.4262	0.0274*	0.5612	0.0133	0.7252	0.0439*
	Local efficiency	0.8754	0.0076	0.7547	0.0029	0.9744	0.0001
	Clustering coeff.	0.8754	0.0041	0.6528	0.0073	0.7932	0.0172
Functional binary	Degree	0.3012	0.0504	0.4708	0.0281*	0.2772	0.2743**
	Global efficiency	0.3012	0.0504	0.4708	0.0281*	0.2772	0.2743**
	Local efficiency	0.4155	0.0270	0.4935	0.0194	0.4530	0.1471*
	Clustering coefficient	0.4155	0.0264	0.4935	0.0193	0.4530	0.1434*

Supplementary Table 1. FDR corrected p-values and effect size quantified by Cohen's f^2 of the factors of the linear mixed effects (LME) model fitting each global network metric. In the p_{FDR} columns * stands for statistical significance ($p_{FDR}<0.05$); in the Cohen's f^2 columns, asterisks represent small (*), medium (**) or large (***) effect size according to convention ($f^2 >0.02$, $f^2 >0.15$, $f^2 >0.35$, respectively).

		Control			TgF344-AD		
**Connectome**	**Network metric**	**p_{FDR}**	**Cohen's f^2**	**R^2**	**p_{FDR}**	**Cohen's f^2**	**R^2**
FD-weighted	Strength	0.4110	0.0734**	0.0687	0.0462*	0.1076**	0.1328
	Local efficiency	0.4110	0.0695**	0.0652	0.0803	0.0849**	0.1547
	Clustering coefficient	0.4110	0.0655**	0.0615	0.0462*	0.1412**	0.0873
Structural binary	Degree	0.4110	0.0345**	0.1272	0.0462*	0.0168	0.1440
	Global efficiency	0.4110	0.0345**	0.1272	0.0462*	0.1683**	0.1440
	Local efficiency	0.7274	0.0051*	0.1831	0.0420*	0.2352**	0.1904
	Clustering coefficient	0.7274	0.0051*	0.1831	0.0420*	0.2352**	0.1904

Supplementary Table 2. Effect of age on the network metrics in each of the groups. FDR corrected p-values, effect size quantified by Cohen's f^2 and R^2 of the linear mixed effects (LME) model fitting each global network metric in control and transgenic animals independently. In the p_{FDR} columns * stands for statistical significance ($p_{FDR}<0.05$); in the Cohen's f^2 columns, asterisks represent small (*), medium (**) or large (***) effect size according to convention ($f^2 >0.02$, $f^2 >0.15$, $f^2 >0.35$, respectively). Group models were evaluated only in the case of significant effect of the interaction between group and age in the LME model fitted to the whole cohort.

		Timepoint									
		**1** (5 months)		**2** (8 months)		**3** (11 months)		**4** (15 months)		**5** (18 months)	
**Connectome**	**Network metric**	p_{FDR}	η^2	p_{FDR}	η^2	p_{FDR}	η^2	p_{FDR}	η^2	p_{FDR}	η^2
FA-weighted	Strength	0.0371*	0.5462***	0.1277	0.2889***	0.9578	-0.0664	1.0000	-0.0667	0.5474	0.0556*
	Global efficiency	0.0566	0.3099***	0.2863	0.0722**	0.9578	-0.0616	0.2637	0.1333**	0.5474	0.0556*
	Local efficiency	0.1261	0.1303**	0.2863	0.0377*	0.9578	-0.0616	0.0994	0.2889***	0.5474	0.0056
	Clustering coefficient	0.0371*	0.4210***	0.1646	0.2056***	0.9578	-0.0664	0.5802	0.0222*	0.5474	0.0556*
FD-weighted	Strength	0.1068	0.1838***	0.1277	0.2599***	0.9578	-0.0696	0.0333*	0.4525***	0.5474	-0.0486
	Global efficiency	0.1261	0.1302**	0.0542	0.5625***	0.9578	-0.0696	0.1022	0.2599***	0.5474	-0.0486
	Local efficiency	0.0971	0.2130***	0.1277	0.2889***	0.9578	-0.0472	0.0333*	0.4889***	0.5474	-0.0486
	Clustering coefficient	0.0848	0.2437***	0.2863	0.1117**	0.9578	0.0344*	0.0333*	0.4889***	0.6056	-0.0611
Structural binary	Degree	0.0371*	0.3824***	0.2863	0.0543*	0.9578	-0.0712	0.5802	-0.0049	0.5474	-0.0153
	Global efficiency	0.0371*	0.3824***	0.2863	0.0543*	0.9578	-0.0712	0.5802	-0.0049	0.5474	-0.0153
	Local efficiency	0.0371*	0.4210***	0.2863	0.0377*	0.9578	-0.0552	0.5802	0.0080	0.5474	0.0556*
	Clustering coefficient	0.0371*	0.4210***	0.2863	0.0377*	0.9578	-0.0552	0.5802	0.0080	0.5474	0.0556*
Functional weighted	Strength	1.556	0.1380**	0.3144	0.0222*	0.9578	-0.0472	1.0000	-0.0642	0.5474	0.1167**
	Global efficiency	0.1068	0.2488***	0.4589	-0.0167	0.9578	-0.0264	1.0000	-0.0667	0.5474	0.0056
	Local efficiency	0.1989	0.0905**	0.2863	0.0377*	0.9578	-0.0616	1.0000	-0.0660	0.5474	0.0292*
	Clustering coefficient	0.3254	0.0114*	0.2863	0.0543*	0.9578	-0.0664	1.0000	-0.0642	0.5474	-0.0333
Functional binary	Degree	0.1156	0.2214**	0.7000	-0.0568	0.9578	-0.0586	1.0000	-0.0568	0.5474	0.0423*
	Global efficiency	0.1156	0.2214**	0.7000	-0.0568	0.9578	-0.0586	1.0000	-0.0568	0.5474	0.0423*
	Local efficiency	0.5698	-0.0677	0.6655	-0.0512	0.9578	-0.0136	1.0000	-0.0667	0.5474	-0.0333
	Clustering coefficient	0.5698	-0.0677	0.6655	-0.0512	0.9578	-0.0136	1.0000	-0.0667	0.5474	-0.0333

Supplementary Table 3. Comparison between network metrics in control and transgenic groups. FDR corrected p-values, effect size quantified by η^2 of the Kruskal-Wallis statistic. In the p_{FDR} columns * stands for statistical significance (p_{FDR}<0.05); in the η^2 columns, asterisks represent small (*), medium (**) or large (***) effect size according to convention (η^2 >0.01, η^2>0.06, η^2>0.14, respectively).

		Group		Metric		Group x metric	
**Connectome**	**Network metric**	**p_{FDR}**	**Cohen's f^2**	**p_{FDR}**	**Cohen's f^2**	**p_{FDR}**	**Cohen's f^2**
FA-weighted	Strength	0.0203*	0.3774***	0.0006*	0.5495***	0.0223*	64.1640***
	Global efficiency	0.4643	0.0835*	0.1778	0.1069*	0.4909	23.1250***
	Local efficiency	0.7994	0.0210*	0.8542	0.0029	0.7634	0.0409*
	Clustering coefficient	0.1525	0.2020**	0.0015	0.3170***	0.1622	55.3820***
FD-weighted	Strength	0.0098	0.3072**	0.0007	0.5465***	0.0016	10.5991***
	Global efficiency	0.0783	0.1382*	0.0035	0.2527**	0.1261	5.0143***
	Local efficiency	0.0098	0.3007**	0.0005	0.7049***	0.0167	15.0485***
	Clustering coefficient	0.0051	0.4168***	1.97e-5	0.9083***	0.0065	18.2783***
Structural binary	Degree	0.0051	0.6597***	1.6e-5	0.7300***	0.0065	140.7851***
	Global efficiency	0.0051	0.6597***	1.6e-5	0.7301***	0.0065	674.7025***
	Local efficiency	0.0098	0.7585***	4.4e-6	0.7568***	0.0064	2530.3059***
	Clustering coefficient	0.0098	0.7585***	4.4e-6	0.7568***	0.0064	565.0728***
Functional weighted	Strength	0.0098	0.1780**	0.1097	0.1401	0.0150	56.0604***
	Global efficiency	0.0149	0.1518**	0.0677	0.2013**	0.018	43.9520***
	Local efficiency	0.0124	0.1607**	0.0458*	0.1765**	0.0167	49.6405***
	Clustering coefficient	0.1242	0.1298*	0.1097	0.1159*	0.1417	38.0199***
Binary weighted	Strength	0.7363	0.0312*	0.3502	0.0727*	0.7107	0.0126
	Global efficiency	0.7363	0.0312*	0.3502	0.0727*	0.7107	0.1067*
	Local efficiency	0.4036	0.0337*	0.5046	0.0540*	0.3740	29.4767***
	Clustering coefficient	0.4036	0.0336*	0.5046	0.0536*	0.3740	3.1351***

Supplementary Table 4. FDR corrected p-values and effect size quantified by Cohen's f^2 of the factors of the linear mixed effects (LME) model fitting the cognitive outcome (number of total trials) as a function of group and each global network metric. In the p_{FDR} columns * stands for statistical significance (p_{FDR}<0.05); in the Cohen's f^2 columns, asterisk represent small (*), medium (**) or large (***) effect size according to convention (f^2 >0.02, f^2 >0.15, f^2 >0.35, respectively).

		Control			TgF344-AD		
**Connectome**	**Network metric**	**p_{FDR}**	**Cohen's f^2**	**R^2**	**p_{FDR}**	**Cohen's f^2**	**R^2**
FA-weighted	Strength	0.9976	0.0192	0.5818	0.0018	0.3979***	0.5230
FD-weighted	Strength	0.9976	0.0153	0.5885	0.0019	0.3739	0.5425
	Local efficiency	0.9976	0.0061	0.5849	0.0014	0.4182***	0.5545
	Clustering coefficient	0.9976	0.0080	0.5857	6.54e-5	0.6385***	0.6146
Structural binary	Degree	0.9752	0.0674*	0.5929	5.29e-5	0.6968***	0.6257
	Global efficiency	0.9752	0.0674*	0.5929	5.29e-5	0.6970***	0.6257
	Local efficiency	0.9752	0.0672*	0.5953	1.26e-5	0.8208***	0.6508
	Clustering coefficient	0.9752	0.0672*	0.5953	1.26e-5	0.8208***	0.6508
Functional weighted	Strength	0.0488	0.1753**	0.5982	0.1570	0.1155*	0.4139
	Global efficiency	0.1689	0.1107*	0.5888	0.1088.	0.1498*	0.4383
	Local efficiency	0.1689	0.1110*	0.5780	0.0771	0.1514**	0.4254

Supplementary Table 5. Effect of network metrics in the cognitive outcome (number of total trials) of each group. FDR corrected p-values, effect size quantified by Cohen's f^2 and R^2 of the linear mixed effects (LME) model fitting cognitive outcome as function of network metrics in control and transgenic animals independently. In the p_{FDR} columns * stands for statistical significance ($p_{FDR}<0.05$); in the Cohen's f^2 columns, asterisk represent small (*), medium (**) or large (***) effect size according to convention ($f^2 >0.02$, $f^2 >0.15$, $f^2 >0.35$, respectively). Group models were evaluated only in the case of significant effect of the interaction between group and network metric in the LME model fitted to the whole cohort.

References

Bajic, D., Craig, M. M., Borsook, D., Becerra, L., & Sullivan, R. M. (2016). Probing intrinsic resting-state networks in the infant rat brain, *10*, 1–13.

Hsu, L.-M., Liang, X., Gu, H., Brynildsen, J. K., Stark, J. A., Ash, J. A., … Yang, Y. (2016). Constituents and functional implications of the rat default mode network. *Proceedings of the National Academy of Sciences*, *113*(31), E4541–E4547.

Jenkinson, M., Beckmann, C. F., Behrens, T. E. J., Woolrich, M. W., & Smith, S. M. (2012). FSL. *NeuroImage*, *62*(2), 782–90.

Sierakowiak, A., Monnot, C., Aski, S. N., Uppman, M., Li, T.-Q., Damberg, P., & Brené, S. (2015). Default mode network, motor network, dorsal and ventral basal ganglia networks in the rat brain: Comparison to human networks using resting state-fMRI. *Plos One*, *10*(3), e0120345
